# Factor Structure of Autistic Traits in Children with ADHD

**DOI:** 10.1007/s10803-013-1865-0

**Published:** 2013-06-09

**Authors:** Joanna Martin, Marian L. Hamshere, Michael C. O’Donovan, Michael Rutter, Anita Thapar

**Affiliations:** 1 MRC Centre for Neuropsychiatric Genetics and Genomics, Institute of Psychological Medicine and Clinical Neurosciences, Cardiff University School of Medicine, Heath Park, Cardiff, CF14 4XN UK; 2 MRC Social Genetic and Developmental Psychiatry Centre (SGDP), Institute of Psychiatry, King’s College London, De Crespigny Park, Denmark Hill, London, SE5 8AF UK

**Keywords:** ADHD, ASD, Factor analysis, Neurodevelopment

## Abstract

Attention-deficit hyperactivity disorder (ADHD) and autism spectrum disorder (ASD) often co-occur. Factor analyses of ASD traits in children with and without ASD indicate the presence of social and restrictive–repetitive behaviour (RRB) factors. This study used exploratory factor analyses to determine the structure of ASD traits (assessed using the Social Communication Questionnaire) in children with ADHD. Distinct factors were observed for ‘social’ and ‘rigidity’ traits, corresponding to previous factor analyses in clinical ASD and population samples. This indicates that the split between social-communicative and RRB dimensions is unaffected by ADHD in children. Moreover, the study also finds that there is some overlap across hyperactive-impulsive symptoms and RRB traits in children with ADHD, which merits further investigation.

## Introduction

Attention-deficit hyperactivity disorder (ADHD) and autism spectrum disorder (ASD) show a high rate of symptom overlap, with a substantial proportion of individuals with one of the conditions also meeting diagnostic criteria for the other (Rommelse et al. 2010). Although historically, diagnostic manuals have not allowed for a joint diagnosis of ADHD and ASD, with an ASD diagnosis trumping and excluding a diagnosis of ADHD (DSM-IV & ICD-10), the observed co-occurrence has prompted changes to these diagnostic exclusions for the DSM-5. Overlap of the two conditions is found at the diagnostic level and at the level of symptoms below diagnostic thresholds in referred children and in the general population (Grzadzinski et al. 2011; Reiersen et al. 2007; Rommelse et al. 2010; Ronald et al. 2008). Moreover, given the high heritability of both conditions (Faraone et al. 2005; Freitag 2006), it is of note that family and twin studies show co-heritability (Lichtenstein et al. 2010; Lundström et al. 2011; Mulligan et al. 2009a; Nijmeijer et al. 2009; Taylor et al. 2012).

In recent years, interest has been growing in exploring the overlap of ADHD and ASD, in terms of associated clinical comorbidities, neuropsychological deficits (e.g. executive functioning) and shared genetic susceptibility. Given the time and cost of performing in-depth interviews and observational diagnostic assessments of ASD, such as the Autism Diagnostic Interview-Revised (ADI-R) and Autism Diagnostic Observation Schedule (ADOS) (Lord et al. 1989, 1994), many studies have relied on questionnaires to measure autistic traits. The Social Communication Questionnaire (SCQ; formerly known as the Autism Screening Questionnaire, ASQ) is a parent-rated questionnaire of ASD behaviours (Berument et al. 1999; Rutter et al. 2003). It is based on the ADI-R (Lord et al. 1994) and has been found to agree well with it on a diagnostic level, although only adequately on an item-by-item basis (Bishop and Norbury 2002). The SCQ is widely used as a screening tool or quantitative measure of autistic traits in samples of children with suspected ASD (Eaves et al. 2006), children with ADHD (Kochhar et al. 2011; Kröger et al. 2011; Mulligan et al. 2009a) or other psychopathology (Pine et al. 2008; Towbin et al. 2005) and also in the general population (Mulligan et al. 2009b). Children diagnosed with ADHD score higher on the SCQ than their siblings or typically developing controls (Kochhar et al. 2011; Mulligan et al. 2009a).

Like the ADI-R, the SCQ can be divided into subscales for the three DSM-IV and ICD-10 diagnostic sub-domains of autistic symptoms: social-interaction deficits, communication problems and restrictive–repetitive behaviours (RRBs). There is a growing body of evidence suggesting that dividing ASD symptoms into these sub-domains is meaningful (Happé and Ronald 2008); although the behaviours occur together frequently, both phenotypic and genetic correlations between them are only moderate, suggesting that the three sub-domains are to some extent clinically and genetically separable (Ronald et al. 2006a, b). Nevertheless, the sub-domains appear to cluster more than expected by chance (Ronald et al. 2006b). In a recent population-based twin sample, the authors tested whether different molecular genetic variants, assessed genome-wide, predicted social and ‘non-social’ (i.e. RRB) traits, separately (Ronald et al. 2010). The study did not find evidence of these clinical domains being separate at a molecular genetic level, although only 1–2 genome-wide genetic variants were found to be nominally associated with either sub-domain (without allowing for multiple testing), and these did not replicate in an independent clinical sample of children diagnosed with ASD. These non-significant results are arguably due to the study’s relatively small sample size for a genome-wide association study that requires multiple testing (ranging from N = 372–436 in the high and low trait comparison groups), making it unclear whether the same genetic variants are involved in these sub-domains. Therefore, more familial and molecular genetic studies in larger samples are needed to clarify the extent to which these social and RRB sub-domains are aetiologically related. Interestingly, the overlap of ADHD with ASD symptoms has been demonstrated to occur within all three of these sub-domains, though RRBs have been found to be less frequent than social and communication deficits in children with ADHD (Rommelse et al. 2011).

Factor analyses of autistic traits in clinical ASD and community samples, using a variety of ASD measurement tools, generally indicate that multiple factors account for the observed covariance structure of ASD symptoms and traits (Happé and Ronald 2008; Mandy and Skuse 2008). Likely due to differences in study design, there is little agreement in terms of the specific factors and their composition. However, nearly all factor analytic studies derive at least one factor related to social-communicative features and a separate factor related to ‘non-social’ behaviour or RRBs (Mandy and Skuse 2008). To date, there has been no published factor analysis of autistic traits in a group of children with ADHD and it is not yet known whether the presence of ADHD affects the nature of autistic symptoms in some manner.

### Aims of the Study

The main aim of this study was thus to explore the structure of autistic traits (as measured by the SCQ) in a clinical sample of children with ADHD, to determine whether this structure was similar to that found in samples of children with ASD and those from the general population. It was hypothesised that social-communicative traits and RRBs would be accounted for by separate, albeit correlated, factors.

A secondary aim was to explore the relationship between ADHD symptoms and ASD traits in a combined exploratory factor analysis. One previous study suggests that the core diagnostic criteria of ADHD and ASD do not overlap (i.e. ADHD and ASD symptoms load on separate sets of factors) in a general population sample of school children (Ghanizadeh 2010). However, although deemed as common conditions, ADHD and ASD have prevalence rates of <5 % in general population samples, with wide variability in reported prevalence rates in different geographic regions (Polanczyk et al. 2007; Rutter 2005). Therefore, it would be valuable to determine whether there is any overlap in ADHD and ASD symptomatology in a clinical sample of children diagnosed with ADHD (and thus having higher rates of individual symptom presence than the average child in the population).

## Methods

### Sample

Children aged 5–18 were referred to the study by clinicians from Child and Adolescent Psychiatry or Paediatric clinics in Wales and across the UK. Inclusion criteria were a current diagnosis of DSM-IV or DSM-III-R ADHD, confirmed by a research diagnostic interview (Angold et al. 1995). Exclusion criteria for the original ADHD genetic study (Stergiakouli et al. 2012) were presence of a clinician’s diagnosis of ASD, Tourette’s syndrome, psychosis, epilepsy, bipolar disorder, brain damage or any other neurological or genetic disorder. The sample consisted of 821 children who met inclusion criteria and had data on the SCQ. Ethical approval for the study was obtained from the North West England and Wales Multicentre Research Ethics Committees. Parents and children aged 16 years and older provided written informed consent and children under 16 provided assent.

### Measures

Attention-deficit hyperactivity disorder was assessed using the Child and Adolescent Psychiatry Assessment (CAPA), a research diagnostic interview undertaken by trained psychologists with the children’s parents, which has well-established high test–retest reliability and construct validity (Angold and Costello 2000; Angold et al. 1995). Parents were asked about presence of each of the 18 ADHD symptoms from the DSM-IV/ICD-10 and about impairment of functioning. The interviewers were supervised weekly by a child psychiatrist and inter-rater reliability for ADHD sub-type research diagnosis, assessed using 60 cases, was perfect (κ = 1.0). Pervasiveness of symptoms, necessary for a DSM-IV diagnosis, was assessed through questionnaires (DuPaul or Conner’s teacher rating scale (Conners et al. 1998; DuPaul 1981)) sent to the school or the Child ADHD Teacher Telephone Interview (ChATTI (Holmes et al. 2004)).

The Social Communication Questionnaire (SCQ) is a 40-item parent-rated questionnaire of autistic traits, with good validity in differentiating children with ASD from those with other learning difficulties and a high correlation with the ADI-R that it is based on (Berument et al. 1999; Rutter et al. 2003). Item 1 of the SCQ is a language screening question and is not included in calculating an overall total of autistic traits. Parents responded to each question by marking ‘yes’ or ‘no’ on the form. Items were re-coded to indicate presence or absence of the autistic behaviour. The 39 items were divided into the three sub-domains of autistic symptoms, as defined by the diagnostic symptoms stipulated by the DSM-IV and ICD-10; there were 20 items classed as ‘social-interaction deficits’, 10 as ‘communication deficits’, 8 as ‘restricted and repetitive behaviours (RRBs)’ and one item (item 18) was unclassified.

Full-scale IQ was assessed with the Wechsler Intelligence Scale for Children (WISC-III & WISC-IV), using all ten subtests (Wechsler 1992, 2003). Comorbid problems were assessed using the CAPA (Angold et al. 1995).

### Factor Analysis

To test the main hypothesis, an exploratory factor analysis (EFA) was performed on the 39 SCQ items and the results were compared with a previous factor analysis of the SCQ in a sample of children with ASD and other psychiatric problems (Berument et al. 1999). The secondary aim of the study was addressed by adding in the 18 ADHD symptoms into the EFA model; this analysis is henceforth referred to as the combined SCQ-ADHD analysis.

Cases with any missing data in either analysis were excluded (SCQ analysis: 97 with 1/39, 52 with 2/39 and 112 with >2/39 items missing; combined SCQ-ADHD analysis: 117 with 1/57, 55 with 2/57 and 115 with >2/57 items missing), leaving a complete data set of N = 560 for the SCQ analysis, and N = 534 for the combined SCQ-ADHD analysis. Children included in the analyses did not differ from those with missing data in terms of gender, age at assessment, family socioeconomic status, severity of their ADHD symptoms or presence of oppositional defiant disorder, conduct disorder or anxiety. They did differ in terms of IQ, with children with missing data having lower IQ than those included (82.4 vs. 84.6, *p* = 0.045 for children in the SCQ analysis and 82.3 vs. 84.7, *p* = 0.019 for those in the combined SCQ-ADHD analysis). All variables were dichotomous (symptom presence/absence). For each analysis, bivariate associations were calculated as tetrachoric correlations. The tetrachoric correlation matrices were not positive definite, so a smoothing algorithm was applied to the correlation matrix (using the R command: tetrachoric (“data”, smooth = T)). Visual inspection of the matrices showed extremely high correlation (tetrachoric correlation = 0.95) of SCQ items 24 “when he/she was 4–5 did he/she nod his/her head to mean *yes*?” and 25 “when he/she was 4–5 did he/she shake his/her head to mean *no*?”, therefore item 25 was dropped from further analyses.

For each analysis, the correlation matrix was analysed using exploratory factor analysis (EFA) to find the minimum residual (minres) solution. This solution was deemed most appropriate given that the assumption of multivariate normality was not fulfilled due to dichotomous variables and the tetrachoric correlation matrices being not positive definite. Choice of number of factors was based on a combination of theory (based on previous literature) and points of inflection on the scree plots. These methods were used in conjunction to maximise variance explained, while maintaining a parsimonious and theory-driven approach towards conceptualising the target construct. For plausible alternative solutions, patterns of correlations across the factor scores were also examined to ascertain the pattern of association across the derived measured factors. The EFA solution was rotated using an oblique rotation (oblimin) as non-independence of the underlying factors was hypothesised (Matsunaga 2010). For ease of interpretation, factor loadings below 0.2 are not shown. All analyses were performed in R.

### Factor Scores

To test whether IQ, age, gender or presence of intellectual disability (ID; IQ < 70) had any effects on the results, factor scores were calculated for each analysis. For each factor, a weighted average score for each individual was calculated using all the loadings from the pattern matrices as weights. Pearson correlations were calculated for each factor score with age and IQ. T-tests were used to compare factor scores in (1) boys relative to girls and (2) children with ID relative to those without ID.

## Results

### ADHD Sample

Demographic information for the sample can be found in Table 1. Additionally, the prevalence of individual ADHD symptoms ranged from 69.9 to 96.6 %. The prevalence of individual SCQ items ranged from 7.9 to 67.3 %.

**Table 1:** Sample demographic information

Variable	Range	Mean	SD
Age at assessment	5–18	10.4	3.0
Total ADHD symptoms	7–18	15.1	2.5
Total SCQ score	0–35	12.8	6.7
IQ score	46–124	84.6	13.9

	N	%
Gender		
Male	473	84.5
Female	87	15.5
Presence of ID		
ID (IQ < 70)	63	11.9
Family socioeconomic status		
Low	259	50.5
Medium	187	36.5
High	67	13.1
ADHD diagnosis subtype		
DSM IV combined subtype	405	72.3
DSM IV predominantly inattentive subtype	28	5.0
DSM IV predominantly hyperactive-impulsive subtype	60	10.7
DSM III-R only	67	12.0
ODD or CD diagnosis		
DSM-IV ODD diagnosis	234	44.5
DSM-IV CD diagnosis	103	18.6
Anxiety or depression diagnosis		
Any DSM-IV anxiety diagnosis^a^	42	7.8
Any DSM-IV depression diagnosis^a^	3	0.6

*SCQ* social communication questionnaire, *ID* intellectual disability, *ADHD* attention deficit hyperactivity disorder, *ODD* oppositional defiant disorder, *CD* conduct disorder

^a^2 children met criteria for a diagnosis of both anxiety and depression

### SCQ Analysis

The scree plot for the EFA of the SCQ items showed two points of inflection: occurring after 3 and 8 factors, explaining 43.8 and 62.2 % of the variance, respectively. A 3-factor solution was chosen on the basis of this information combined with theory (the DSM-IV & ICD-10 distinguish between three sub-domains of ASD and the majority of previous factor analytic studies suggest that three factors are meaningful (Mandy and Skuse 2008)). Factor 1 was modestly correlated with factors 2 and 3 but factors 2 and 3 were uncorrelated (see Table 2). Table 3 shows the pattern matrix of loadings for the rotated solution, indicating which items load highly on each ASD sub-domain (based on DSM-IV).

**Table 2:** Factor correlations for both EFA analyses

	SCQ EFA			SCQ-ADHD EFA	
	Factor 1—social	Factor 2—rigidity	Factor 3—non-verbal communication	Factor 1—social	Factor 2—rigidity/hyperactivity
SCQ EFA					
Factor 2—rigidity	0.43*				
Factor 3—non-verbal communication	0.37*	−0.05			
SCQ-ADHD EFA					
Factor 1—social	0.98*	0.39*	0.54*		
Factor 2—rigidity/hyperactivity	0.47*	0.97*	−0.16*	0.39*	
Factor 3—inattentiveness	0.13*	0.17*	−0.12*	0.11*	0.24*

*SCQ* social communication questionnaire, *EFA* exploratory factor analysis, *ADHD* attention deficit hyperactivity disorder

* Correlation is significant at the 0.01 level (2-tailed)

**Table 3:** Pattern matrix of loadings for factor analysis of SCQ items

SCQ item	DSM-IV sub-domains	Factor 1—social	Factor 2—rigidity	Factor 3—non-verbal communication
34-joins in social games (4/5)	COM	0.78		
37-positive response to other children (4/5)	SOC	0.77		
39-plays imaginative games with others (4/5)	COM	0.77		
36-interest in other children (4/5)	SOC	0.76		
27-reciprocates smiles (4/5)	SOC	0.73		
30-wants others to join in (4/5)	SOC	0.69		
29-shares things (4/5)	SOC	0.67		
40-plays cooperatively (4/5)	SOC	0.67		
38-attention without name called (4/5)	SOC	0.66		
33-range of facial expressions (4/5)	SOC	0.63		
35-pretend play (4/5)	COM	0.61		0.25
31-comforts others (4/5)	SOC	0.60		
28-shows things to engage interest (4/5)	SOC	0.57		0.30
26-looks at faces (4/5)	SOC	0.45	0.28	
2-talks to be friendly	COM	0.30		0.23
10-appropriate facial expressions	SOC	0.21		
8-repeats things exactly	COM		0.81	
4-uses odd phrases	COM		0.80	
17-repetitive complicated movements	RRB		0.72	
13-interested in parts not whole	RRB		0.72	
7-invents words/phrases	COM		0.68	
16-unusual movements	RRB		0.65	
5-inappropriate questions	SOC		0.61	
9-has rituals	RRB		0.55	
11-uses other’s hand as tool	SOC		0.54	
12-unusual interests	RRB		0.54	
15-unusual sensory interests	RRB		0.54	
6-pronoun reversal	COM		0.50	
14-unusually intense interests	RRB		0.48	
18-self-injures	–	0.20	0.43	
19-always carries specific object around	RRB		0.37	
3-can have a conversation	COM	0.30	0.31	0.27
20-has friends	SOC		0.22	
32-uses gestures with sounds/words (4/5)	SOC			0.71
24-nods head (4/5)	SOC			0.67
22-points to show things (4/5)	SOC	0.27		0.66
23-uses gestures (4/5)	SOC			0.66
21-copies people’s actions (4/5)	COM	0.29		0.40

*COM* communication sub-domain, *SOC* social-interaction sub-domain, *RRB* restrictive and repetitive behaviours sub-domain. To aid interpretation, loadings of < 0.2 are not presented

Factor 1 was comprised of items regarding social-interaction and communication skills and was labelled the ‘social’ factor. Similarly to a previous factor analysis of the SCQ in children with ASD and other psychiatric problems (Berument et al. 1999), this ‘social’ factor is comprised primarily of a similar set of social-interaction items. Factor 2 in the current analysis was comprised of all the RRB items as well as a few of the social and communication items and was labelled as the ‘rigidity’ factor. The majority of items comprising the ‘rigidity’ factor are all of those that constituted two separate factors labelled ‘abnormal language’ and ‘stereotyped behaviour’ in the previous EFA of the SCQ in children with ASD and other psychiatric problems (Berument et al. 1999). Factor 3 contained items to do with gesturing and was labelled the ‘non-verbal communication’ factor. In the factor analysis by Berument et al. (1999), these items clustered with the majority of the social-interaction items in the ‘social’ factor.

### Combined SCQ-ADHD Analysis

Next, the 18 ADHD symptoms and 38 SCQ items were analysed together. The scree plot showed two points of inflection: occurring after 3 and 5 factors, explaining 35.3 and 44.6 % of the variance, respectively. The range of factor inter-correlations for the 3-factor solution (see Table 2) was lower than that for the 5-factor solution (r = −0.08–0.46, *p* = 0.23–*p* < 0.001), indicating greater coherence between the derived factors of the 3-factor solution relative to the 5-factor solution. On the basis of this information and the ‘parsimony principle’ (Kline 2010) a 3-factor solution was chosen. All three factors were modestly correlated (see Table 2). The rotated factor loadings are shown in Table 4, indicating which items load on which ASD sub-domains (based on DSM-IV). Factor 1 was very similar to the ‘social’ factor of the SCQ analysis, as can be seen both by its composition and correlation with this factor (see Table 2). Likewise, factor 2 of the combined SCQ-ADHD analysis was very similar to the ‘rigidity’ factor of the SCQ analysis, with the addition of the majority of the hyperactive-impulsive ADHD symptoms. Factor 3 comprised the inattentive ADHD symptoms and one of the hyperactive symptoms. These factors were labelled ‘social’, ‘rigidity/hyperactivity’ and ‘inattentiveness’, respectively.

**Table 4:** Pattern matrix of loadings for factor analysis of SCQ items and ADHD symptoms

	DSM-IV sub-domains	Factor 1—social	Factor 2—rigidity/hyperactivity	Factor 3—inattentiveness
SCQ 30-wants others to join in (4/5)	SOC	0.75		
SCQ 39-plays imaginative games with others (4/5)	COM	0.73		
SCQ 34-joins in social games (4/5)	COM	0.72		
SCQ 27-reciprocates smiles (4/5)	SOC	0.70		
SCQ 28-shows things to engage interest (4/5)	SOC	0.69		
SCQ 35-pretend play (4/5)	COM	0.69		
SCQ 29-shares things (4/5)	SOC	0.64		
SCQ 22-points to show things (4/5)	SOC	0.63		
SCQ 36-interest in other children (4/5)	SOC	0.62	0.25	
SCQ 31-comforts others (4/5)	SOC	0.62		
SCQ 37-positive response to other children (4/5)	SOC	0.61	0.29	
SCQ 38-attention without name called (4/5)	SOC	0.58	0.22	
SCQ 40-plays cooperatively (4/5)	SOC	0.57	0.28	
SCQ 33-range of facial expressions (4/5)	SOC	0.56		
SCQ 26-looks at faces (4/5)	SOC	0.50	0.28	
SCQ 32-uses gestures with sounds/words (4/5)	SOC	0.50		
SCQ 21-copies people’s actions (4/5)	COM	0.46		
SCQ 3-can have a conversation	COM	0.42	0.28	
SCQ 2-talks to be friendly	COM	0.41		
SCQ 24-nods head (4/5)	SOC	0.34		
SCQ 20-has friends	SOC	0.30		
SCQ 23-uses gestures (4/5)	SOC	0.22		
SCQ 10-appropriate facial expressions	SOC	0.21		
SCQ 13-interested in parts not whole	RRB		0.74	
SCQ 8-repeats things exactly	COM		0.69	
SCQ 17-repetitive complicated movements	RRB		0.66	
SCQ 4-uses odd phrases	COM		0.66	
SCQ 7-invents words/phrases	COM		0.65	
SCQ 11-uses other’s hand as tool	SOC		0.63	
SCQ 16-unusual movements	RRB		0.62	
SCQ 12-unusual interests	RRB		0.58	
SCQ 9-has rituals	RRB		0.56	
SCQ 15-unusual sensory interests	RRB		0.56	
SCQ 18-self-injures	–		0.51	
ADHD IMP 4-talking excessively	IMP		0.50	
SCQ 5-inappropriate questions	SOC		0.49	
SCQ 14-unusually intense interests	RRB		0.49	
ADHD HYP 3-running excessively	HYP		0.47	
SCQ 6-pronoun reversal	COM		0.41	
ADHD HYP 5-often noisy	HYP		0.39	0.36
ADHD HYP 4-on the go	HYP		0.37	
SCQ 19-always carries specific object around	RRB		0.37	
ADHD HYP 1-fidgeting	HYP		0.37	0.29
ADHD IMP 1-waiting for turn	IMP		0.32	0.27
ADHD IMP 2-blurting out answers	IMP		0.26	
ADHD IMP 3-interrupting	IMP		0.23	0.23
ADHD IN 1-sustaining attention	INA			0.74
ADHD IN 7-making careless mistakes	INA			0.72
ADHD IN 2-following instructions	INA			0.71
ADHD IN 8-forgetful	INA			0.69
ADHD IN 9-organisational difficulty	INA			0.65
ADHD IN 3-avoiding mental effort	INA			0.62
ADHD IN 6-not listening	INA			0.60
ADHD IN 4-easily distracted	INA			0.55
ADHD IN 5-losing things	INA			0.44
ADHD HYP 2-remaining seated	HYP		0.29	0.41

*COM* communication sub-domain, *SOC* social-interaction sub-domain, *RRB* restrictive and repetitive behaviours sub-domain, *IMP* impulsive symptoms, *HYP* hyperactive symptoms, *INA* inattentive symptoms. To aid interpretation, loadings of < 0.2 are not presented

It can be seen from Table 2 that all the factors from the SCQ analysis are significantly correlated with those from the combined SCQ-ADHD analysis. The majority are positively correlated, with the exception of the SCQ ‘non-verbal communication’ factor with the SCQ-ADHD ‘rigidity/hyperactivity’ and ‘inattentiveness’ factors (those comprising ADHD symptoms), which were negatively correlated.

### Correlates of Factor Scores

Age at assessment was negatively correlated with the ‘rigidity’ factor of the SCQ analysis and the ‘rigidity/hyperactivity’ factor of the combined SCQ-ADHD analysis and positively correlated with the ‘non-verbal communication’ factor of the SCQ analysis. In other words, younger children showed higher rates of RRBs and hyperactive-impulsive behaviours, whereas older children showed more of some of the social-communication ASD traits. Correlation results are displayed in Table 5. The factor scores from both analyses were negatively correlated with IQ (*p* < 0.05), except for the ‘inattentiveness’ factor from the combined SCQ-ADHD analysis, which only showed a trend towards a negative correlation (*p* = 0.09).

**Table 5:** Factor score correlations with age and IQ

Factor	Age	IQ
SCQ EFA		
Factor 1—social	0.01	−0.21**
Factor 2—rigidity	−0.13**	−0.12**
Factor 3—non-verbal communication	0.12**	−0.13**
SCQ-ADHD EFA		
Factor 1—social	0.05	−0.21**
Factor 2—rigidity/hyperactivity	−0.20**	−0.10*
Factor 3—inattentiveness	−0.03	−0.08

* Correlation is significant at the 0.05 level (2-tailed)

** Correlation is significant at the 0.01 level (2-tailed)

Children with ID (IQ < 70) had higher factor scores for the first two factors of either analysis (*p* < 0.05), i.e. the ‘social’, ‘rigidity’ and ‘rigidity/hyperactivity’ factors. These results are displayed in Table 6. In terms of gender, boys tended to have higher scores than girls (*p* < 0.05) on the ‘social’ factor of the SCQ analysis. There were no gender differences for the other factors (see Table 6).

**Table 6:** Association of factor scores with gender and presence of ID

Factor	Gender	Mean (SD)	t	*p*	Presence of ID	Mean (SD)	t	*p*
SCQ EFA								
Factor 1—social	F	0.25 (0.19)	−2.18	0.03	No ID	0.28 (0.22)	−3.95	<0.001
	M	0.30 (0.23)			ID	0.40 (0.25)		
Factor 2—rigidity	F	0.40 (0.22)	0.25	0.80	No ID	0.37 (0.24)	−2.81	0.01
	M	0.39 (0.25)			ID	0.46 (0.27)		
Factor 3—non-verbal communication	F	0.32 (0.28)	−0.25	0.80	No ID	0.32 (0.28)	−1.51	0.13
	M	0.33 (0.29)			ID	0.38 (0.32)		
SCQ-ADHD EFA								
Factor 1—social	F	0.24 (0.20)	−1.61	0.11	No ID	0.26 (0.21)	−3.49	<0.001
	M	0.28 (0.22)			ID	0.37 (0.23)		
Factor 2—rigidity/hyperactivity	F	0.50 (0.19)	−0.39	0.70	No ID	0.49 (0.21)	−2.44	0.01
	M	0.51 (0.22)			ID	0.57 (0.23)		
Factor 3—inattentiveness	F	0.89 (0.18)	0.54	0.59	No ID	0.88 (0.19)	−1.36	0.17
	M	0.88 ( 0.19)			ID	0.91 (0.17)		

*ID* intellectual disability (IQ < 70)

## Discussion

In children with ADHD, the results of the SCQ factor analysis indicate that items tapping into social deficits and RRBs constitute mainly separate factors, with various items for communication deficits clustering with one or the other factor. In comparison to a previous factor analysis of the SCQ in children with ASD and other psychiatric problems (Berument et al. 1999), the largest factor is comprised of social-interaction items, whereas items concerning RRBs constitute a separate factor, which in this previous EFA were sub-divided into two separate factors labelled ‘abnormal language’ and ‘stereotyped behaviour’. Although there are differences in which specific items are included in the different factors in the current analysis compared to the one by Berument and colleagues (1999), the similarities are striking.

Moreover, the results suggest that the factor structure of autistic traits (measured by the SCQ) in children with ADHD is comparable to previous reports of factor analyses of autistic measures in children with ASD and the general population showing separate social and ‘non-social’ or RRB factors (Happé and Ronald 2008; Mandy and Skuse 2008). It is important to note though, that despite these separate clusters of items, these factors (in particular the ‘social’ and ‘rigidity’ factors) are correlated, indicating that they are not independent of each other. Although the symptoms reported here are not at diagnostic levels, these results also support the growing body of evidence for changing ASD diagnostic criteria from a triad (social-interaction deficits, communication problems and RRBs) to a dyad (social-communication deficits and RRBs) of symptom types in the DSM-5 (Frazier et al. 2012; Mandy et al. 2012).

The second analysis, which explored whether ADHD symptoms and items of the SCQ group together or form separate factors, found that the two factors of items of social-communicative deficits from the SCQ factor analysis (i.e. the ‘social’ and ‘non-verbal communication’ factors) combined into a single ‘social’ factor and the hyperactive-impulsive symptoms tended to group with RRB items in a ‘rigidity/hyperactivity’ factor, with a separate factor for inattentive ADHD symptoms. Although there are minor differences in which items load more strongly on the specific factors in this analysis, compared to the analysis of the SCQ items only, the division of the ASD traits into ‘social’ and ‘rigidity’ factors appears unaffected by including ADHD symptoms in the analysis.

The observed division of the ADHD symptoms into separate factors with hyperactive-impulsive and inattentive symptoms is well-supported in the ADHD literature (Willcutt et al. 2012). Indeed, as with social-communicative symptoms relative to RRBs (Ronald et al. 2006a, b, 2010), there is evidence suggesting that hyperactive-impulsive and inattentive symptoms show some level of genetic heterogeneity and specificity in addition to substantial genetic overlap (Greven et al. 2011). It is of particular interest that the hyperactive-impulsive ADHD items clustered with the RRB items and we can only speculate as to why this might be the case. This finding requires further investigation and replication in other samples.

There is growing evidence that ADHD and ASD are each the extreme end of a continuum rather than being distinct categories (Constantino and Todd 2003; Larsson et al. 2011; Levy et al. 1997). Given this, the division of ADHD into two dimensional scales of inattentive and hyperactive-impulsive symptoms and of ASD into social-communication and RRB dimensions has important implications for classification of developmental problems, investigating the aetiology of these traits and understanding the high heritability and co-heritability of the two disorders (Faraone et al. 2005; Freitag 2006; Lichtenstein et al. 2010).

Although a 3-factor solution for the combined SCQ-ADHD analysis is believed to be the optimal solution, an alternative was to choose a less parsimonious 5-factor solution (full results available from the first author). This solution bears many similarities to the main analyses. The primary factor is composed of a highly similar set of items to the SCQ ‘social’ and the SCQ-ADHD 3-factor ‘social’ factors and is very highly correlated with these (Pearson correlations of 0.99 and 0.98, respectively). Similarly, the second factor of the 5-factor SCQ-ADHD solution is very highly correlated with the ‘rigidity’ SCQ factor (0.995) and the ‘rigidity/hyperactivity’ factor (0.98) of the SCQ-ADHD 3-factor analysis. The third factor appears to correspond to the ‘inattentiveness’ factor of the SCQ-ADHD 3-factor analysis (correlation = 0.98). The fourth factor contains primarily hyperactive and impulsive ADHD symptoms and the fifth factor corresponds to the ‘non-verbal communication’ factor of the SCQ analysis (correlation = 0.96). The key point about the 5-factor solution is that the ASD items come out separately to the ADHD symptoms (i.e. it has three factors of ASD items corresponding to the SCQ analysis and two separate factors for ADHD symptoms). Such a solution is in line with the previous exploratory factor analysis of core ADHD and ASD diagnostic criteria in a population sample of school children (Ghanizadeh 2010). However, it is important to consider competing factor solutions and further studies are needed to clarify the extent of the overlap of RRBs and hyperactive-impulsive ADHD symptoms. It would also be worth exploring the factor structure of ADHD and ASD symptoms in children diagnosed with ASD. One small study (N = 65) has attempted to do this, and although they found distinct factors for ASD and ADHD, they do not consider competing models and provide no clear justification for the choice of a 2-factor solution (Ghanizadeh 2012).

Although children with ID (IQ < 70) tend to be excluded from studies of ADHD and sometimes also of ASD, these children were included in the present analyses (N = 63). Given the high association of lower IQ and higher rates of ID in these neurodevelopmental conditions (Frazier et al. 2004; Voigt et al. 2006), IQ is not statistically separable from neurodevelopmental problems (Dennis et al. 2009) and the deliberate recruitment of children without ID may bias representativeness of such samples. Indeed, analysis of factor scores in relation to IQ showed that the factor scores were negatively correlated with IQ, indicating that children with the most severe symptom profiles were likely to score lower on the IQ test. There is also evidence that children with ADHD and comorbid ID do not differ in their ADHD profile to those with ADHD without ID (Ahuja et al. 2013; Antshel et al. 2006). A complete re-analysis of the data excluding the children with ID shows no marked differences to the pattern of observed results (available from first author upon request).

Given that ADHD and ASD are developmental conditions, it is not surprising that some of the factor scores showed associations with age. There appeared to be no effect for age for the primary ‘social’ factors, whereas the ‘rigidity’ and ‘rigidity/hyperactivity’ factor scores decreased with age and the ‘non-verbal communication’ factor scores increased with age. Although it is well-established that hyperactivity and impulsivity decrease with age (Willcutt et al. 2012), it is less clear why older children would struggle more on the items comprising the ‘non-verbal communication’ factor, unless this is related to parental recall of items from when the children were aged 4–5.

In terms of gender, boys had higher scores than girls on the ‘social’ factor of the SCQ analysis but boys and girls did not differ on the other factor scores. Given that there is a high ratio of boys to girls in samples of children with ADHD and ASD, it is reasonable that boys with ADHD are more likely to have higher ASD scores than girls, although it is unclear why this is the case only for the social difficulties. Limiting the analysis to boys-only makes no difference to the pattern of observed results (details available upon request).

## Study Limitations and Conclusions

The results of this study need to be considered in light of several limitations. Data on ADHD and ASD were derived from different types of instruments. Whilst ADHD symptoms were measured using a diagnostic interview completed with parents (Angold et al. 1995), ASD traits were measured using a questionnaire measure (Rutter et al. 2003). Future studies will need to examine whether the pattern of results is affected by the type of instrument used.

Although the relatively large sample size was a strength of the current study, it was not sufficiently large enough to analyse stratified age groups. Also, parental recall of their children’s behaviour (particularly at ages 4–5) might have been different for adolescents. The findings of this study relate to clinic children diagnosed with ADHD and it is possible that the observed association of hyperactive-impulsive items with RRBs might not be evident in children with lower levels of such traits, as has been suggested by one other study (Ghanizadeh 2010).

However, despite these caveats, the study contributes novel findings to the growing body of literature exploring the overlap of ADHD and ASD. The results highlight that there are distinct dimensions of social-communicative difficulties and RRBs in children diagnosed with ADHD, supporting such a finding in children with ASD and in the general population (Mandy and Skuse 2008). This finding further implies that the presence or absence of ADHD does not affect the manifestation of social-communicative difficulties and RRBs in children. The results also support the switch from a triad to a dyad of diagnostic symptom dimensions in the DSM-5 (Frazier et al. 2012; Mandy et al. 2012). The suggestion that hyperactive-impulsive traits may be linked with RRBs is an intriguing one and requires replication and further study, both in clinically referred children and in the general population.
